# The Ingestion of 62 Magnetic Beads by a Two-Year-Old Child: A Case Report of a Novel Approach for Retrieval

**DOI:** 10.7759/cureus.64541

**Published:** 2024-07-14

**Authors:** Muhammad Aishat, Omayr M Irshad, Faris M Shurafa, Sharif Mohamed

**Affiliations:** 1 Medicine, Texas College of Osteopathic Medicine, The University of North Texas Health Science Center, Fort Worth, USA; 2 Anesthesiology, University of Texas Medical Branch at Galveston, Galveston, USA; 3 Public Health, University of Houston, Houston, USA

**Keywords:** pediatric, case report, ingestion, swallowed foreign body, magnetic beads

## Abstract

The incidence of foreign body ingestion in young children has been increasing over the past couple of years. Although less than 1% of ingested foreign bodies require surgical intervention, the clinician’s awareness should be heightened when the ingested body has a magnetic component. Potential complications of multiple magnetics include intestinal necrosis, perforation, ileus, and sepsis.

This case study highlights the clinical presentation, surgical methods, anesthetic considerations, and the need for pediatric intensive care unit (PICU) admission in a two-year-old female child who ingested magnetic beads. The paper presents the patient's history, diagnosis, and surgical procedure, including the use of a novel magnetic apparatus to locate the foreign bodies.

Clinicians should have a heightened sense of caution when treating children who have ingested multiple magnetic foreign bodies due to potential gastrointestinal complications and increased morbidity. The case describes the use of a novel approach in utilizing a pacemaker magnet to identify any remaining ingested magnetic foreign bodies in the bowel.

## Introduction

Foreign body ingestion is a frequent occurrence in young children, particularly those aged five and under. According to data from the National Poison Data System (NPDS), foreign bodies/toys/miscellaneous objects in children under the age of five comprised 6.51% of indexed America Poison Center cases in 2021 [1]. Notably, the incidence of magnetic foreign body misingestion (MFBM) has steadily increased, as reported in single-center studies at multiple institutions [2,3]. In response to this, the United States Consumer Product Safety Commission (CPSC) issued a warning on the hazardous nature of powerful magnets found in toys [4]. From 2003 to 2006, a report was published by the Centers for Disease Control and Prevention regarding 20 instances of complicated magnet ingestion in children ranging from 10 months to 11 years old. Among these cases, 75% resulted in bowel perforations, and 20% suffered from generalized peritonitis [5].

In this case study, we demonstrate the clinical presentation, the surgical methods utilized to remove the magnetic foreign bodies using a magnetic apparatus with fluoroscopy, anesthetic considerations, and the necessity of pediatric intensive care unit (PICU) admission following surgery. This manuscript was prepared following the CARE guidelines (https://www.care-statement.org).

## Case presentation

**Figure 1:**
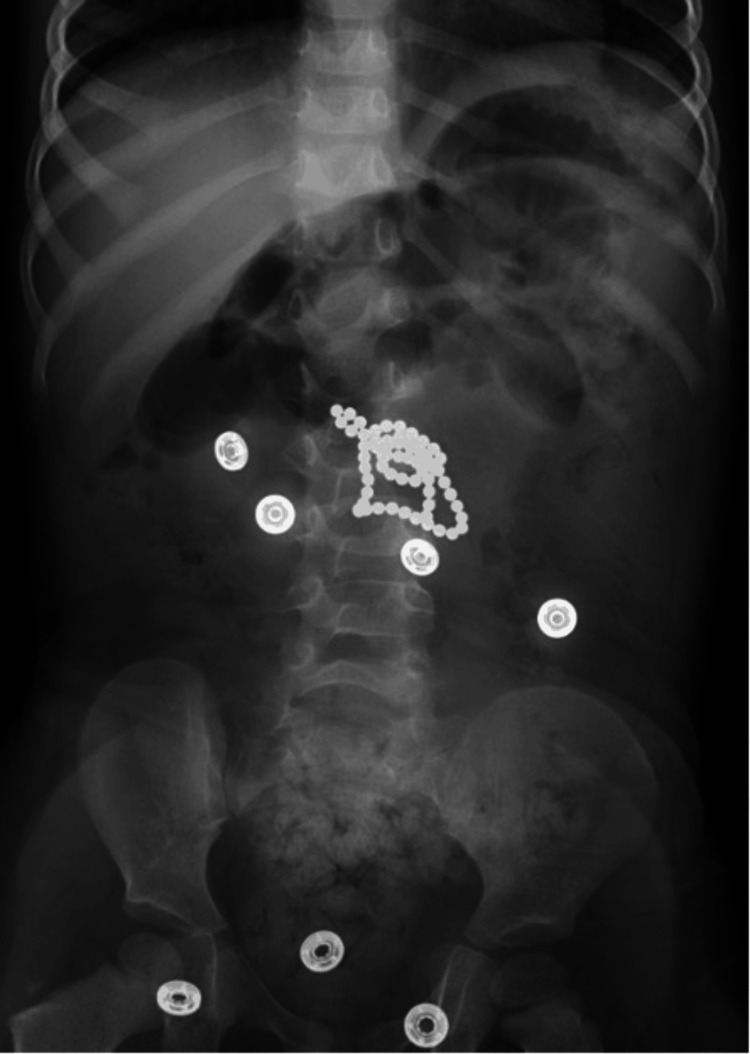
Abdominal X-ray of Foreign Body and Small Bowel Obstruction

**Figure 2:**
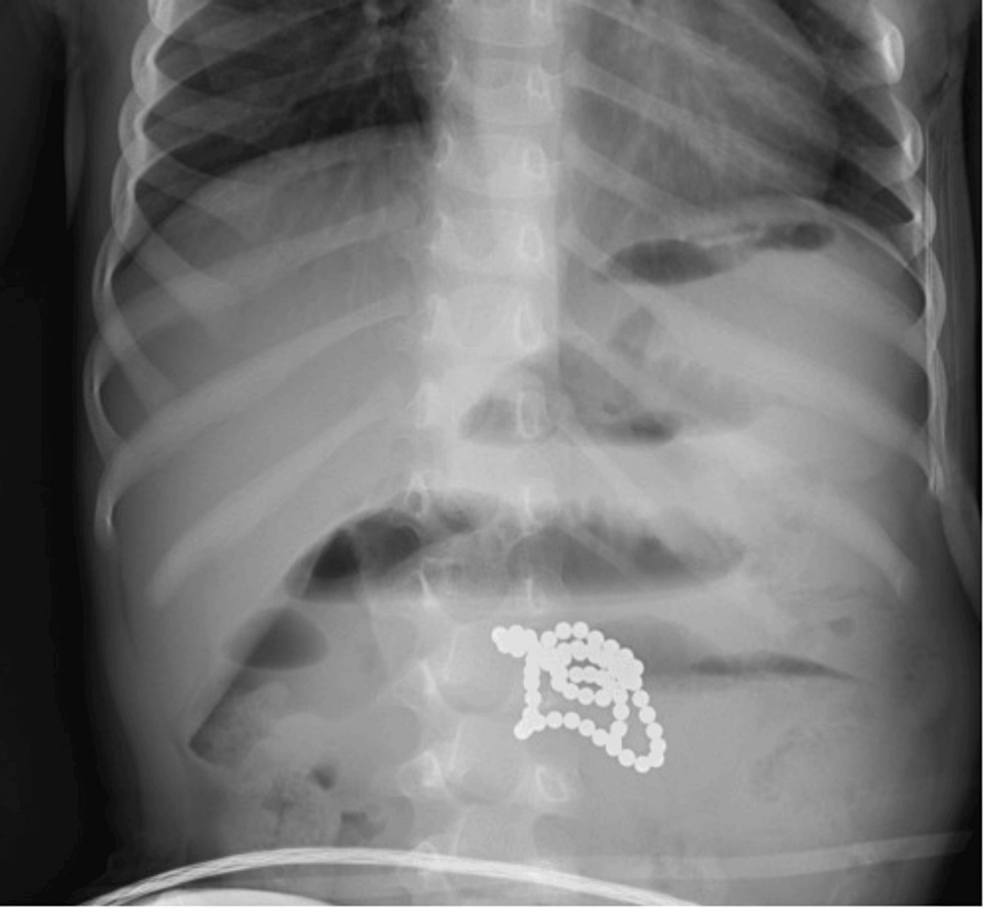
Abdominal X-ray With Multiple Dilated Bowel Loops

A two-year-old female patient with no significant past medical history presented on Saturday, March 12, 2022, to a satellite emergency department (ED) with her parents, who reported that she had been experiencing changes in behavior since the morning of the previous day. Per the parents, the patient was found on Friday morning sleeping next to a pile of “white puke”. Upon waking up, she vomited hourly non-bilious, non-bloody emesis. Later in the day, the emesis turned brown in color, followed by a “bile green.” She did not tolerate any solid food but drank apple juice and an electrolyte drink. The patient was diagnosed with influenza A at an urgent care clinic and sent home with oseltamivir and supportive instructions. However, the patient continued to vomit hourly, leading the parents to bring her to the ED. The patient’s mother stated that she believed the patient may have ingested magnetic beads since she noticed some of them had gone missing that day. Abdominal X-ray in the ED indicated a stable foreign body within the mid-abdomen with resultant small bowel obstruction (Figure 1). A repeated X-ray indicated stable multiple dilated loops of bowel measuring up to 3.6 cm (Figure 2). The dilated loops of the bowel were proximal to the radiopaque foreign body in the mid-abdomen. The patient was transferred to the main hospital for an emergency laparotomy and anticipated extended PICU stay.

In the operating room, the patient underwent decompression of the stomach with an orogastric tube prior to intubation, as well as additional fluid resuscitation, additional line placements, and a preoperative caudal block. The patient was then intubated without complications. A nasogastric tube was placed as well as a 10 French (Fr) Foley catheter. Fluoroscopy was used to visualize the greatest concentration of the foreign object (magnetic beads) and the site was appropriately marked. A 4 cm midline incision was made and carried down to the fascia without complication. The small bowel was found to be eviscerated and the distal bowel was noted to have evidence of a perforation with magnetic beads present.

A small bowel resection, measuring approximately 5 cm, was performed at the mid to distal ileum. Posterior wall Lembert sutures were placed to approximate the bowel. The posterior wall lumen was closed with a 4-0 polydioxanone suture (PDS) in a running fashion. The anterior layer was closed with a 4-0 PDS using the Connell stitch pattern. The lumen was patent, and the bowel appeared viable. Proximally, in the distal jejunum-proximal ileum area, there was a bulging subserosal hemorrhage noted along 15 cm of bowel with bleeding from the mesentery, which was subsequently removed. The underlying bowel appeared normal, and the serosa was repaired with 3-0 silk Lembert sutures. The bowel was run more proximally and two small enterotomies from the beads were noted with healthy surrounding bowel. These were repaired with 3-0 silk sutures. One area separate from the concentration of the majority of beads in the proximal jejunum was noted. These beads required a small 5 mm enterotomy to remove them from the bowel lumen by milking them outward. This enterotomy was closed with 3-0 silk.

Throughout the case, visualization of the magnets was limited due to their size and presence in the bowel lumen. To overcome this, a novel magnetic apparatus was utilized in conjunction with fluoroscopy to better locate the beads. This device consisted of simply placing a pacemaker magnet inside a sterile surgical bag. Given that the foreign magnetic beads are composed of neodymium magnets, which are the strongest naturally occurring magnets in the world, attracting them was not difficult when using our surgical magnetic apparatus. By utilizing this approach, the likelihood of remnant beads in the peritoneal cavity was decreased and a different modality for detection of these beads was proven to be effective.

**Figure 3:**
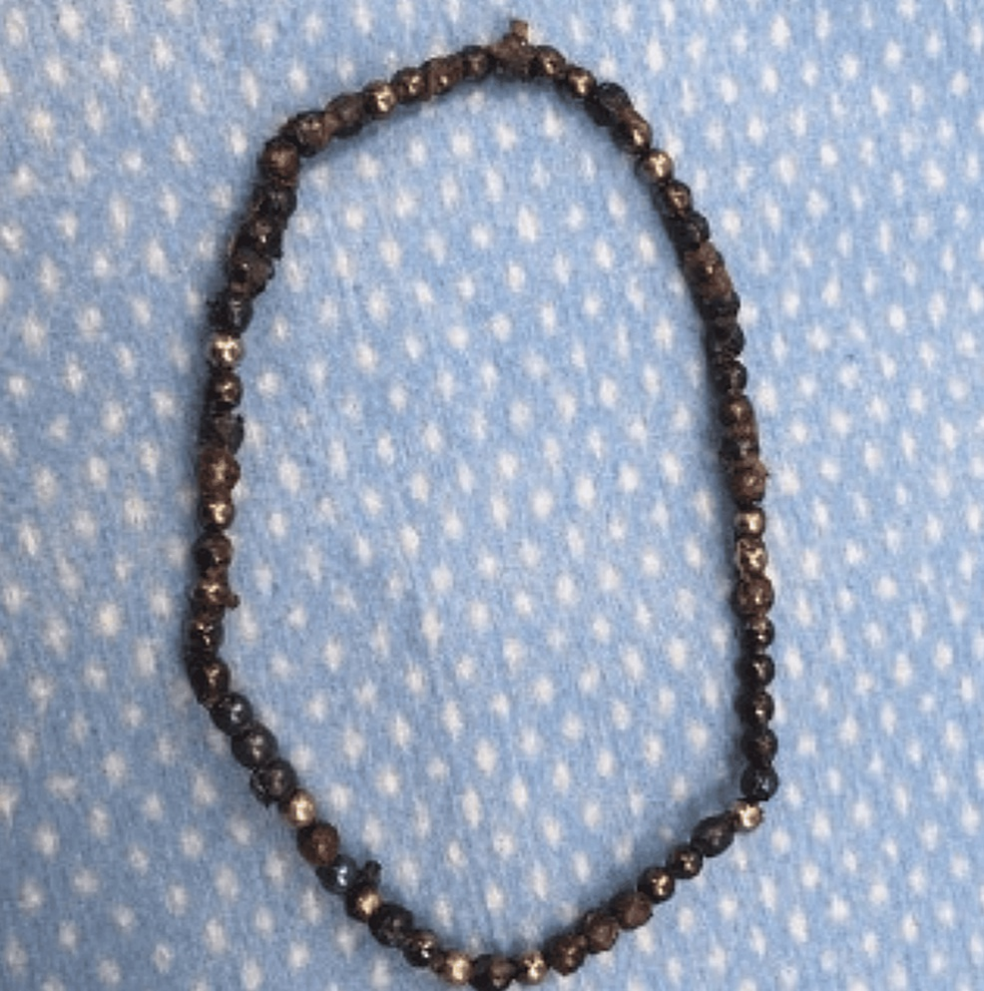
Removal of 62 Beads

The total number of beads removed from the gastrointestinal tract totaled 62 (Figure 3). Final inspection of the abdomen using palpation of the bowel, the magnetic device, and fluoroscopy revealed no additional beads present. An incidental appendectomy was performed with 2-0 polyglactin 910 (Vicryl; Ethicon, Inc., Somerville, USA) suture to ligate the base followed by inversion of the appendiceal stump with 3-0 silk. The bowel was run from the ligament of Treitz to the ileocecal valve and no additional injuries were noted. Adequate peak pressures were noted when the bowel was returned to the abdomen. The patient was then extubated and transferred to the post-anesthesia care unit (PACU) in critically stable condition. The beads were reported to the CPSC.

## Discussion

Management of ingested foreign bodies includes allowing natural discharge, emergent endoscopic removal, or surgical laparotomy [6]. Most foreign bodies in the digestive tract, roughly 80-90%, are discharged spontaneously without any medical intervention [7]. Approximately 10-20% of cases require endoscopic removal [7]. Surgical interventions typically comprise less than 1% of ingested foreign body cases [8]. Although most foreign body ingestions are asymptomatic and result in unaided discharge, several objects have an increased risk of causing adverse outcomes. These objects include sharp objects, batteries, and multiple magnets [9]. In a review of multiple single-center studies, it appears that ingestion of multiple magnets predisposes to several gastrointestinal pathologies more so than ingestion of a single magnet or intermittent ingestion of multiple magnets [2,3]. When two or more magnets are ingested together or shortly apart, the magnetic interaction can lead to compression of the luminal wall, as demonstrated in a case where two magnets in the gastric body were attracted to three magnets in the duodenum [10]. Compression of the luminal wall can result in ischemic necrosis, which ultimately may progress to intestinal perforation, small bowel obstruction, volvulus, hemorrhage, peritonitis, and fistulas [4,11,12]. Our case of ingested foreign magnets was an emergency where surgery should not be delayed. The case had inherent risks with aspirations, bowel dysfunction, and gastric distention, which led us to perform a rapid sequence intubation. Our methodology in this case included a novel apparatus, specifically the placement of an anesthetic placement magnet in a sterile bag. This technique serves as a crucial tool during our surgery for precise foreign body localization. In cases where multiple magnets were ingested with enough time between each magnet, the attraction of each individual magnet has a negligible attraction toward the other magnets, which increases the likelihood of spontaneous discharge [3]. Notably, magnetic beads and toys of this nature are readily accessible throughout the United States. The lack of adequate warning labels or comprehensive safety information accompanying many of these products is concerning. In light of these safety concerns, we advocate for the reporting of such items to the CPSC (cpsc.gov), as it is for the well-being of individuals, particularly children, who may come into contact with these objects.

## Conclusions

This case presented a novel approach in which a novel magnetic apparatus using a pacemaker magnet was used to identify and retrieve magnetic foreign bodies in a pediatric patient. Healthcare providers must exercise heightened caution when dealing with patients suspected of ingesting magnetic foreign bodies, as multiple magnets can lead to severe complications, including bowel necrosis, necessitating urgent intervention. Given the widespread availability of powerful magnetic toys in the market without proper warnings, reporting these products to the CPSC is essential. Furthermore, raising awareness among the public about the dangers of magnetic toys is crucial to prevent accidents and protect individuals from harm. It should also be noted that in this case and many others, the child necessitated management at a hospital with the capacity to operate on children as well as safely manage their post-operative stay. In this case, the hospital stay was limited to a site with a PICU appropriate to ensure the best outcomes for the patient.
